# Post-Spaceflight (STS-135) Mouse Splenocytes Demonstrate Altered Activation Properties and Surface Molecule Expression

**DOI:** 10.1371/journal.pone.0124380

**Published:** 2015-05-13

**Authors:** Shen-An Hwang, Brian Crucian, Clarence Sams, Jeffrey K. Actor

**Affiliations:** 1 Department of Pathology and Laboratory Medicine, University of Texas Medical School at Houston, Houston, Texas, United States of America; 2 Division of Biomedical and Environmental Sciences, NASA Johnson Space Center, Houston, Texas, United States of America; 3 Space and Clinical Operations Division, NASA Johnson Space Center, Houston, Texas, United States of America; Texas Tech University, UNITED STATES

## Abstract

Alterations in immune function have been documented during or post-spaceflight and in ground based models of microgravity. Identification of immune parameters that are dysregulated during spaceflight is an important step in mitigating crew health risks during deep space missions. The *in vitro* analysis of leukocyte activity post-spaceflight in both human and animal species is primarily focused on lymphocytic function. This report completes a broader spectrum analysis of mouse lymphocyte and monocyte changes post 13 days orbital flight (mission STS-135). Analysis includes an examination in surface markers for cell activation, and antigen presentation and co-stimulatory molecules. Cytokine production was measured after stimulation with T-cell mitogen or TLR-2, TLR-4, or TLR-5 agonists. Splenocyte surface marker analysis immediate post-spaceflight and after *in vitro* culture demonstrated unique changes in phenotypic populations between the flight mice and matched treatment ground controls. Post-spaceflight splenocytes (flight splenocytes) had lower expression intensity of CD4^+^CD25^+^ and CD8^+^CD25^+^ cells, lower percentage of CD11c^+^MHC II^+^ cells, and higher percentage of CD11c^+^MHC I^+^ populations compared to ground controls. The flight splenocytes demonstrated an increase in phagocytic activity. Stimulation with ConA led to decrease in CD4^+^ population but increased CD4^+^CD25^+^ cells compared to ground controls. Culturing with TLR agonists led to a decrease in CD11c^+^ population in splenocytes isolated from flight mice compared to ground controls. Consequently, flight splenocytes with or without TLR-agonist stimulation showed a decrease in CD11c^+^MHC I^+^, CD11c^+^MHC II^+^, and CD11c^+^CD86^+^ cells compared to ground controls. Production of IFN-γ was decreased and IL-2 was increased from ConA stimulated flight splenocytes. This study demonstrated that expression of surface molecules can be affected by conditions of spaceflight and impaired responsiveness persists under culture conditions *in vitro*.

## Introduction

Since the beginning of space travel, there have been reports of reoccurring/opportunistic infections during and post-spaceflight, likely resulting from immune suppression associated with mission parameters. Cosmonauts developed acute respiratory issues after prolonged spaceflight [1], and astronauts on Apollo and Skylab missions experienced increased infections [2, 3]. Published reports documented an increase in reactivation of latent viruses (Epstein-Barr virus, Cytomegalovirus, and Varicella-Zoster virus) pre-, during, and post-spaceflight compared to healthy ground control individuals [4–9]. Multiple factors were cited to alter host immune function of viral control, with stress parameters being the marker of highest correlative value [10, 11]. Viral reactivation is often accompanied by elevated stress hormones, including cortisol, epinephrine, and norepinephrine [5, 7, 12, 13]. Additionally, shifts in circulating cytokines towards high levels of IL-4, IL-6, and IL-8 were observed in astronauts found shedding viral particles [4].

Studies on spaceflight impact on immune function have been investigated through analysis of blood leukocytes obtained from astronauts, cells secured from space flown mice and rats, and *in vitro* cell culture systems [14]. Several changes in cell populations have been noted immediately post-spaceflight, including decreased total leukocytes, decreased T-cells (specifically CD4^+^ T-cells), increased monocytes, increased granulocytes, and decreased natural killer cells [15–17]. However, reports of alterations in circulating leukocytes populations isolated from astronauts post-spaceflight are at best inconsistent; other analyses found no changes in lymphocyte or monocytes populations [10, 18–21]. The inconsistency of the data is most likely due to mission specific variables and individual health status.

The observed changes in immune activity post-spaceflight involves both innate (macrophages and NK cells) and adaptive (T-cell) functions. This report represents the first occasion of a detailed analysis of both surface marker expression and response to stimulation parameters that mimic a broad range of pathogen induced activation events. In order to answer these questions, this study focused on mouse splenocyte composition and function immediately post 13 days of spaceflight, utilizing animals that were part of the historic final flight of NASA’s Space Shuttle Program (Space Shuttle Atlantis, STS-135). Innate stimulation of toll-like receptors (TLR), the surface receptors that target molecular pathogenic patterns, examined activation events using agonists employed primarily by bacterial or fungal agents. These included zymosan (TLR-2 agonist), lipopolysacchride (LPS; TLR-4 agonist), and flagellin (TLR-5 agonist).The adaptive stimulation parameters included T-cell stimulation using antibodies to stimulate CD3 (T-cell receptor) and CD28 (T-cell co-receptor), or via mitogen, concanvalin A, to bypass the CD28 co-receptor.

Analysis of mouse splenocytes focused on changes in surface marker expression for T-cells and dendritic cells (DCs), marking a distinction between innate and adaptive immunity. DCs activate upon innate TLR stimulation, leading to increased expression of antigen presentation molecules (MHC I and II) and co-stimulatory molecules (CD86 and 80). Upon cell-cell contact with DCs, CD8^+^ (MHC I) or CD4^+^ (MHC II) T-cells are activated by antigen recognition through the T-cell receptor (CD3 is the signal transduction region) and the co-stimulatory molecule CD28. The goal of these studies was to determine the influence of spaceflight on immune activity known important for translating innate immune responses to long-lasting T-cell hypersensitive activity.

## Materials and Methods

### Flight Information: Subjects and Spaceflight

This study was conducted as a subset of a larger parent flight investigation identified as Commercial Biomedical Test Module-3, designed to determine if administering an experimental agent preflight reduces the loss of bone associated with spaceflight [22]; multiple investigators shared materials collected post flight [23–27]. For the parent study, mice (“flight mice”) were flown onboard Space Shuttle Atlantis, mission STS-135, for approximately 13 days. For this immune-specific sub-study, approximately ½ spleen from six C57BL/6 mice (Charles River, 9 weeks of age at start of flight) were made available immediately following spaceflight. The subjects were flown in a Space Shuttle Animal Enclosure Module (AEM), which was housed in the mid-deck area of the vehicle for the duration of the spaceflight. Spleens from six ground-based control mice, which were handled and housed in a ground-based identical manner (AEM module) to the flight mice were included in the study. The AEM module housing conditions for control mice have been well described [28]. AEM ground control mice received vehicle control diluting agent.

The mice were part of the NASA Ames Research Center’s Biospecimen Sharing Program (http://www.nasa.gov/ames/research/space-biosciences/cbtm-3-sts-135). Flight experiments were designed to investigate effects of microgravity on vascular atrophy in mouse hindlimbs. We are aware that among our six flight mouse subjects, four were treated with a bone-specific medication and two were untreated. For purposes of this article, analysis was conducted on all six flight subjects as a group, without knowledge of treatment, and data were compared to ground AEM control subjects. The individual mice that were drug or placebo treated remained unknown during experimentation; data presented here was not separated according to treatments. To our knowledge, there was no influence of medication on the data collected. Separate analysis of data did not indicate statistical outliers within the combined spaceflight mice, which would indicate affects due to treatment. In light of this, we believe our findings are relevant and important, even in light of inclusion of mice that received the bone loss experimental drug.

### Mouse Splenocytes Processing

Animals were retrieved approximately 1 hour following deorbit, and sacrificed within 3–5 hours post flight, collected on July 21, 2011 at Kennedy Space Center (Cape Canaveral, FL). Mice were euthanized using 4% isoflurane followed by cardiac puncture and exsanguinations [28]. Half of the spleen was processed as previously described [28]. Briefly, the organ was homonenized between glass slides, RBC lysed with ACK buffer (Lonza), washed twice with 1x Cellgro Dulbecco’s Phosphate Buffer Saline (PBS, Mediatech), and cultured in complete DMEM media [Dulbecco’s Modified Eagles Medium (Sigma) supplemented with 2.2g/L sodium bicarbonate, 0.05g/L of HEPES (Sigma), 0.05g/L L-arginine (Sigma), 100 μg/mL penicillin G (Sigma), 50 μg/mL gentamycin sulfate (Sigma) 0.005% 2-mercaptoethanol (Gibco), and 10% fetal bovine serum (Sigma)] at 1x10^6^cells/mL. Processing of splenocytes for *in vitro* analysis occurred within 4 hours post flight. The animal welfare committee at the University of Texas Health Science Center was consulted prior to initiation of experimentation; no protocol was required as samples used were obtained post euthanasia. All NASA activities with these mice were performed according to guidelines stipulated in the Guide for the Care and Use of Laboratory Animals as described by the National Institutes of Health. Experiments were performed after approval by review boards associated with NASA’s standard operating procedures, and as reported for tissue used in recent reports [26, 28].

### Bead Uptake Assay

Splenocytes were cultured with 1.0μm FluoSpheres polystyrene microsphere (Invitrogen) at ratio of 10 beads per 1 cell. At 2 hours, cells were washed with 1x PBS, fixed with 4% paraformaldehyde for 15 minutes on ice, and stored in flow buffer (1% BSA in 1x PBS) for analysis.

### Stimulation Assays

Isolated splenocytes were cultured for 24 hours in media alone or stimulated with 1) anti-CD3 (0.1μg/mL) and anti-CD28 (0.1μg/mL) antibodies (eBiosciences), 2) 2μg/mL Concanavalin A (ConA, Sigma), 3) 200ng/mL lipopolysaccharide (LPS, O111:B4, Sigma), 4) 10μg/mL Zymosan (*Saccharomyces cerevisiae*, Invivogen), or 5) 100ng/mL flagellin (*Salmonella typhimurium*, Invivogen). At 24 hours, supernatants were collected for ELISA and cells collected for staining of surface markers.

### Flow Cytometry

Identification of surface molecule expression by flow cytometric analysis was conducted as previously described [29]. Briefly, samples were blocked by incubating purified anti-CD16/32 (Fc block, BD Biosciences) on ice for 15 minutes. Then, samples were stained with mouse specific T-cell markers or DC markers antibody cocktails in staining buffer (1% BSA in 1xPBS) on ice for 45–60mins. T-cell marker cocktail: CD4-FITC, CD8-PE, CD28-PECy5, CD-25-PECy7, and CTLA-4-APC. DC marker cocktail: H_2_K^b^-FITC, I-A^b^-PE, CD86-PECy5, CD11c-PECy7, and CD80-APC. Stained samples were fixed with 4% paraformaldehyde on ice for 15 mins. Stained and fixed samples were stored in 500μL of staining buffer. Samples were read on Gallios Flow Cytometer (Beckman-Coulter). Analysis was conducted with Cyflogic.

### Cytokine Analysis

Supernatants were analyzed by DuoSet (R&D Systems) according to manufacturer’s protocol as previously described [29–31]. Supernatants were analyzed for inflammatory cytokines (TNF-α, IL-6) and lymphocyte cytokines (IL-2, IFN-γ). Lower limits of detection for the assay were between 16–32 pg/mL.

### Statistics

Splenocytes were collected from 6 mice per group. Flow analysis counted 20,000 events per sample. ELISA analysis was conducted in duplicate or triplicate. Data was analyzed by TwoWay ANOVA followed by Bonferroni post-hoc test. Significance is considered when p<0.05.

## Results

### Splenocyte Population Changes between Flight Mice and Ground Controls

**Fig 1:**
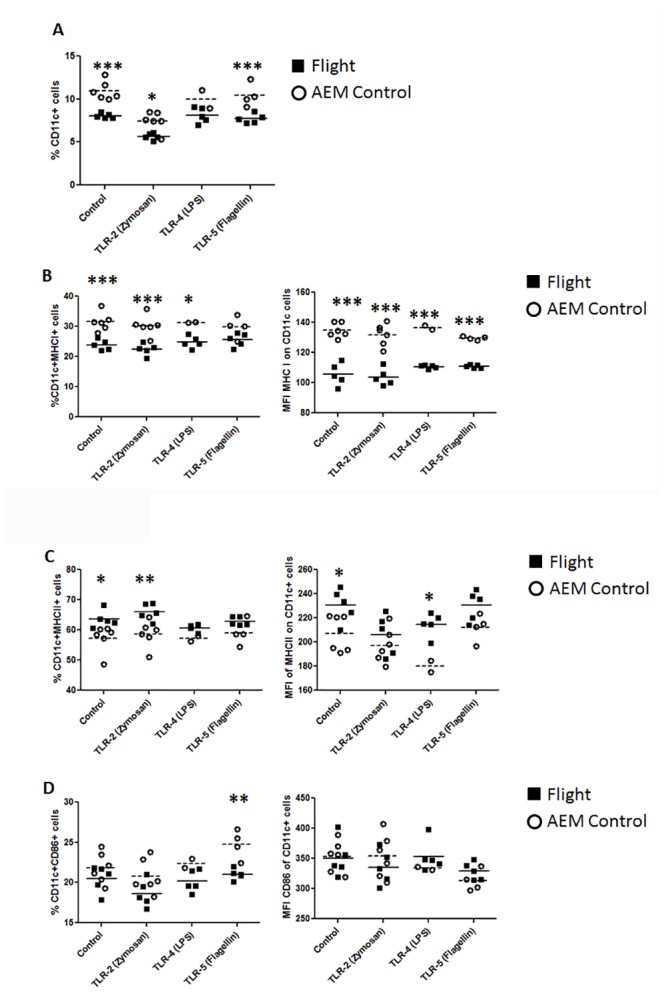
Cell population differences in total splenocytes collected from flight mice. Splenocytes collected immediately post-spaceflight were stained with CD4, CD8, MHC I, MHC II, and CD11c. Analysis was conducted by flow cytometry. (1a) Example of gating strategy between flight mice and ground (AEM; animal enclosure module controls). (1b) Example of positive population composition of each gate identified in 1a from flight mice. (1c) Graph of percent positive and MFI from each marker of splenocytes from flight mice.

Basic differentiation of splenocyte cell populations between flight mice and matched ground controls were completed by flow cytometric analysis. Forward scatter (FS) and side scatter (SS) parameters were noted, with the former measuring size of particle and the latter measuring granularity. Cells were assessed immediately post 13 days in flight, or following short term culture *in vitro*. Distinct changes in total splenocyte population were noted. Native splenocytes collected from spleens immediate upon sacrifice demonstrated two distinct cell populations, based on FS. The flight mice splenocytes selectively demonstrated presence of a third population within gate 3 (G3) with higher FS compared to ground controls (Fig 1a). Fig 1b shows representative dot plots of cell population makeup within each gate identified in Fig 1a. There are clear population differences in cells expressing CD4, CD8, MHC I and MHC II (Fig 1b). Examination of T-cell and antigen presentation cell markers on the different cell populations found that G3 cells contained a higher percentage of CD4^+^ and CD8^+^ cells compared to G1 and G2 populations. The G3 population also had increased surface expression of MHC I compared to G1 and G2 cells (Fig 1c). This indicates that post-spaceflight mice have spleens with slightly higher CD4 and CD8 T-cell populations compared to matched ground controls.

**Fig 2:**
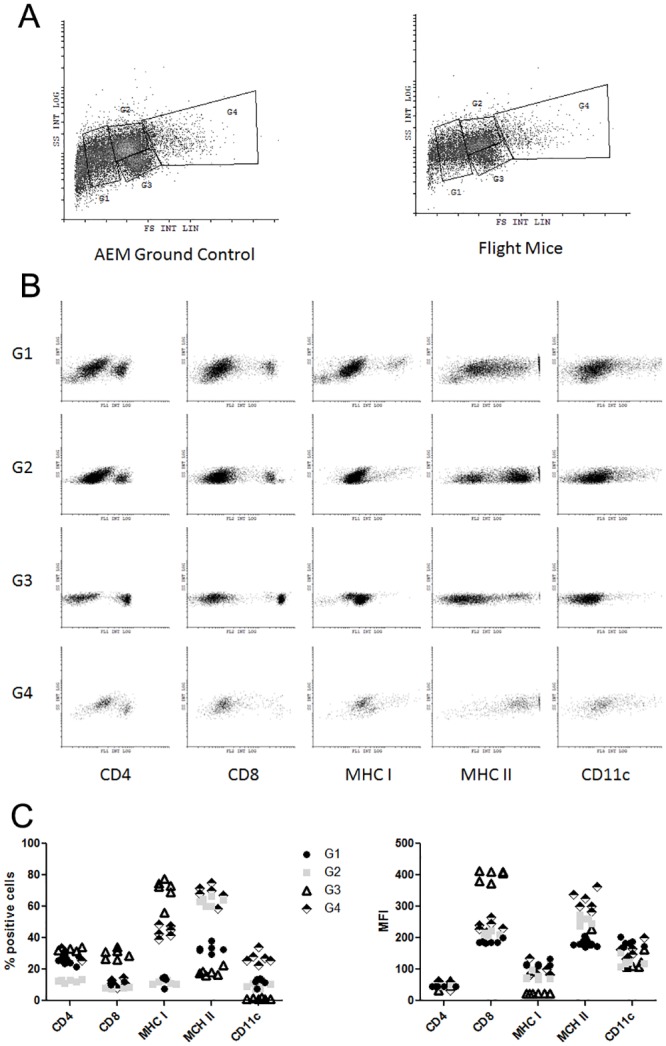
Cell population differences in total splenocytes collected from ground control mice. Splenocytes collected immediately post-sacrifice of ground control mice were stained with CD4, CD8, MHC I, MHC II, and CD11c. Analysis was conducted by flow cytometry. (2a) Example of gating strategy between flight mice and ground controls. (2b) Example of positive population composition of each gate identified in 2a from ground AEM control mice. (2c) Graph of percent positive and MFI from each marker of splenocytes from AEM control mice.

Upon culture for 24 hours in cell media, relative marker expression for the splenocyte population shifted. Cultured splenocytes isolated from ground control mice exhibited an increase in cell density in G3 compared to cultured splenocytes isolated from flight mice (Fig 2a). Examination revealed that G3 population has higher percentage of CD8^+^ and MHC I^+^ cells. It was also noted that G3 population also displayed increased surface expression of CD8^+^ as indicated by increased mean fluorescent intensity (MFI). Cell populations of G2 and G4 display surface marker expression typical of dendritic cells and macrophages (Fig 2b and 2c). These data suggests that *in vitro* culturing of splenocytes from ground control mice enhances relative abundance of CD8 T-cell marker expression compared to splenocytes collected from the mice post-spaceflight.

### Splenocyte Markers Altered Post-Spaceflight

With obvious differences in cell population observed from splenocytes collected from ground control versus flight mice within hours post-spaceflight, detailed examination of surface marker changes were conducted. In addition to changes in overall T-cell and dendritic cell populations, markers important for T-cell activation and proliferation and dendritic cell antigen presentation were also examined (Tables 1 and 2).

**Table 1:** Comparative Splenocyte T-cell Surface Marker Expression Post-Spaceflight.

	% population		p value	MFI		p value
	AEM Control	Flight		AEM Control	Flight	
**CD4**	17.87 (0.6524)	15.85 (1.476)	0.2381	**45.92 (1.151)**	**40.64 (0.5718)**	**0.0021**
**CD4+CD28+**	16.26 (0.6261)	15.21 (1.456)	0.5226	18.15 (0.9947)	19.02 (1.024)	0.558
**CD4+CD25+**	0.7833 (0.057)	0.63 (0.065)	0.1081	**33.62 (5.298)**	**12.47 (0.3354)**	**0.0026**
**CD8**	15.78 (0.9637)	15.45 (1.201)	0.8305	**87.12 (3.247)**	**103.7 (5.853)**	**0.0323**
**CD8+CD28+**	12.42 (0.6122)	14.30 (1.159)	0.1819	15.4 (1.019)	13.94 (1.132)	0.3608
**CD8+CD25+**	0.3867 (0.0451)	0.4050 (0.0381)	0.7624	**57.91 (8.846)**	**13.32 (3.294)**	**0.008**

Data obtained by flow cytometry using labeled antibodies detailed in Materials and Methods. Cells assessed from splenocytes collected within hours post-spaceflight (Flight) mice or animal enclosure module (AEM) ground control mice. Average values given with standard deviation. Bold values represent significant differences and relative p values between groups. MFI; mean fluorescent intensity.

**Table 2:** Comparative Antigen Presentation Cell Marker Expression Post-Spaceflight.

	% population		p value	MFI		p value
	AEM Control	Flight		AEM Control	Flight	
**CD11c**	**13.41 (0.7944)**	**16.2 (0.4225)**	**0.0113**	69.89 (1.076)	67.73 (1.416)	0.251
**MHC I**	43.13 (1.694)	46.01 (0.9846)	0.1722	74.91 (1.755)	72.23 (1.241)	0.2402
**MHC II**	7.43 (0.4261)	7.348 (0.201)	0.8659	**124.5 (7.879)**	**90.07 (3.083)**	**0.0023**
**CD11c+MHC I**	**51.65 (0.8139)**	**57.85 (1.189)**	**0.0015**	60.18 (2.168)	63.75 (1.359)	0.1941
**CD11c+MHC II**	**52.32 (2.029)**	**47.73 (0.465)**	**0.052**	85.86 (1.797)	83.08 (3.03)	0.4478
**CD11c+MHC I/II**	6.763 (0.6634)	8.123 (0.1472)	0.0732	N/A	N/A	

Data obtained by flow cytometry using labeled antibodies detailed in Materials and Methods. Cells assessed from splenocytes collected within 3–5 hours post-spaceflight (Flight) mice or animal enclosure module (AEM) ground control mice. Average values given with standard deviation. Bold values represent significant differences and relative p values between groups. MFI; mean fluorescent intensity. N/A; not assessed.

Analysis of cell populations indicated that the majority of lymphocytes resided within the G2 and G3 gated areas. As such, examination of changes in T-cell populations was restricted to G2 and G3 events. T-cell markers of interest examined are CD4, CD8, CD28 (the second signal for T-cell activation), CD25 (IL-2 receptor), and CTLA-4 (the signal for T-cell anergy). No differences were observed in percent of splenocytes expressing these surface markers between flight and ground control mice. However, several significant changes were observed in the intensity of surface marker expression. Splenocytes from flight mice had a significant decrease in CD4 MFI, with a concurrent increase in CD8 MFI, compared to ground controls. The intensity of CD25 expression in both CD4 and CD8 cells were significantly decreased in splenocytes from flight mice compared to ground controls (Table 1). Little to no CTLA-4 expression was detected from any of the samples (data not shown).

Examination of antigen presentation and co-stimulatory molecules were focused on events occurring in cells gated within G1, G2, and G3. There was a significant increase in population of splenocytes expressing the dendritic cell marker (CD11c) from flight mice compared to ground controls. A similar increase was also observed in dendritic cells (CD11c+) expressing MHC I. However, a significant decrease was detected in dendritic cells co-expressing MHC II from splenocytes isolated from flight mice compared to ground controls (Table 2).

### Post-Spaceflight Splenocytes Increased Ability to Uptake Particles

A central process in innate immunity is the ability to phagocytose. To examine if post-spaceflight conditions affected the ability of splenocytes to uptake particles, splenocytes were incubated with fluorescent beads, and analyzed for bead ingestion conducted by flow cytometry.

**Fig 3:**
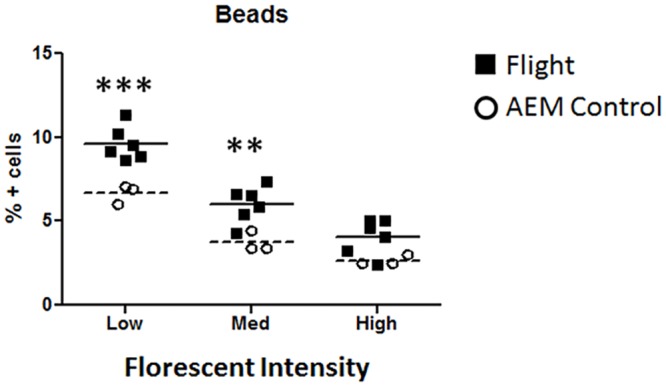
Bead uptake by splenocytes from flight mice and ground controls. Splenocytes were incubate with fluorescent beads and collected for analysis by flow cytometry. Percent positive events were separated by peak mean florescent intensity and labeled as low, med, and high. Data was plotted for each individual mouse. Mean indicated by dotted line (ground AEM control) or solid line (flight mice). *** = p<0.001; **p = <0.01

Three distinct populations for positive bead uptake were detected, and labeled as low, medium, high according to florescent intensity (FI). Cells from G1, G2, and G3 were considered as one population and distinguished only by FI. Splenocytes from flight mice demonstrated a significant increase in percent positive cells at low and medium FI, and a non-significant increase at high FI compared to ground controls (Fig 3).

### Changes in T-cell Surface Molecules upon Stimulation

**Fig 4:**
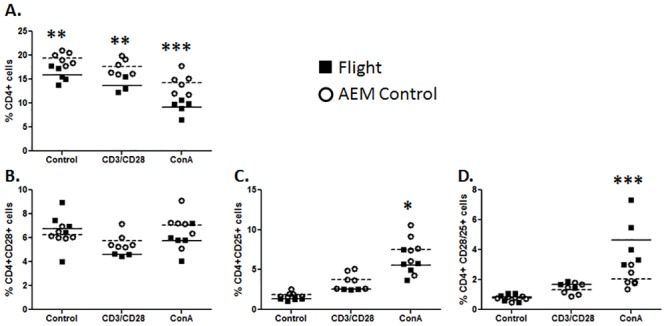
Surface markers expression of CD4^+^ splenocytes. Flight (solid square) or ground AEM control (open circle) splenocytes were cultured in DMEM with 10% FBS for 24 hours with only media (control), anti-CD3/CD28, or ConA. Cells were collected and stained for expression of CD4, CD28, and CD25. (A) CD4 positive cells in total splenocyte population. (B) CD4^+^CD28^+^ cells. (C) CD4^+^CD25^+^ cells. (D) CD4^+^ cells that are also CD28^+^CD25^+^. *** = p<0.001 **p = <0.01; * = p<0.05

Next, changes in T-cells surface marker expression post stimulation with anti-CD3/CD28 antibodies and Concanavalin A (ConA) were examined. The total population of CD4^+^ cells was significantly decreased in splenocytes collected from flight mice in all cultured conditions (media control, CD3/28, or ConA) compared to ground controls. Additionally, stimulation of cultured cells also caused a downward trend of CD4 expressing cells compared to culturing in media control (Fig 4a). There was no significant difference in cell population expressing both CD4 and CD28 (Fig 4b). Stimulation with ConA dramatically increased the CD4^+^CD25^+^ population, with a significant decrease observed in splenocytes from flight mice compared to ground controls (Fig 4c). A similar increase in CD4 cell population co-expressing CD28 and CD25 was observed during ConA stimulation. An unexpected finding was that CD4^+^ splenocytes from flight mice demonstrated and increase in CD28/25 co-expressing cells compared to ground controls (Fig 4d).

**Fig 5:**
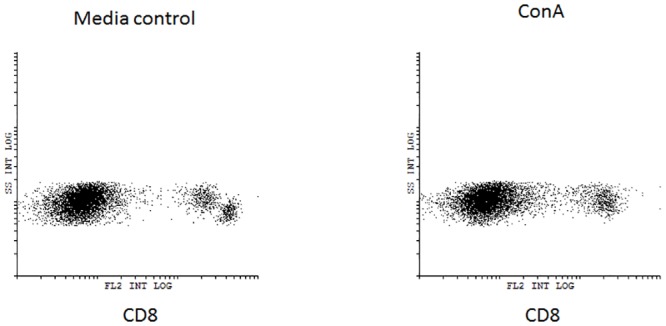
Expression of CD8 on non-activated and ConA activated splenocytes. Flight (solid square) or ground AEM control (open circle) splenocytes were cultured in DMEM with 10% FBS for 24 hours with or without ConA (2μg/mL). Cells were collected and stained for expression of CD8. Representative flow dot graphs of CD8 positive populations after 24 hours culture in media only (left) or in the presence of ConA (right).

Previous observations in our laboratory demonstrated that the action of *in vitro* culturing leads to altered cell population (non-published observation). For example, simple *in vitro* culturing of splenocytes in media alters intensity of CD8 expression. This was now examined in the post-flight group. After 24 hours in cell media, splenocytes, from both flight and ground controls, developed two distinct CD8^+^ cell populations based on mean fluorescent intensity (MFI). The populations were termed CD8lo^+^ and CD8hi^+^. Upon stimulation with ConA for 24 hours, this distinction disappears and most of the CD8^+^ splenocytes are CD8lo^+^ (Fig 5).

**Fig 6:**
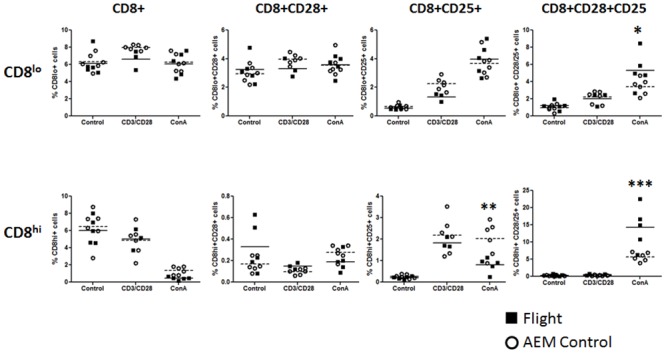
Expression of surface markers of CD8^lo^ and CD8^hi^ splenocytes. Flight (solid square) or ground AEM control (open circle) splenocytes were cultured in DMEM with 10% FBS for 24 hours with only media (control), anti-CD3/CD28, or ConA. Cells were collected and stained for expression of CD8, CD28, and CD25. *** = p<0.001 **p = <0.01; * = p<0.05

Examination of CD8 expression during stimulation is divided between CD8lo and CD8hi populations. There is little if any change in CD8 expression among the media control and CD3/28 and Con A stimulated groups. However, CD8hi expression is very low after stimulation with ConA. No significant differences between splenocytes isolated from flight mice or ground controls in all culture conditions. The exception is the observation of a significant increase in CD25^+^CD28^+^ expression of CD8^+^ T-cells from splenocytes of flight mice compared to ground controls (Fig 6).

### Presentation and Activation Molecule Changes in Splenocytes during TLR Stimulation

Effective immunity against pathogens relies on innate recognition of molecular patterns and activation of antigen presentation. Toll-like receptors (TLR) are well characterized pattern recognition receptors that initiate activation of the innate response. Using agonists for TLR-2 (Zymosan), TLR-4 (LPS), and TLR-5 (flagellin), splenocytes were stimulated for 24 hours and analyzed for surface expression of antigen presentation and co-stimulatory molecules.

**Fig 7:**
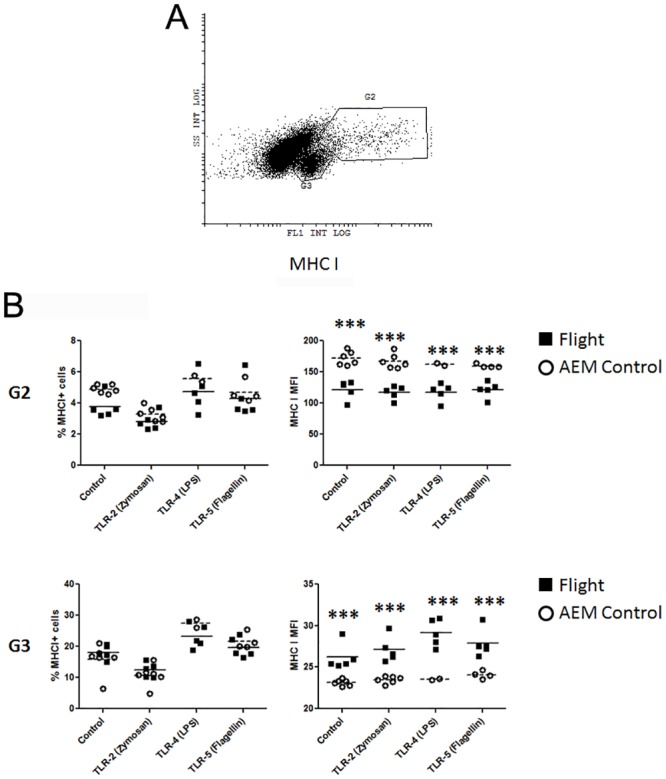
Antigen presentation molecule expression under TLR agonist stimulation. Flight (solid square) or ground AEM control (open circle) splenocytes were cultured in DMEM with 10% FBS with or without TLR agonists for 24 hours. Cells were collected and stained for expression of MHC I (H-2k^b^) and MHC II (I-A^b^). (7a) Representative dot plot of MHC I^+^ populations. (7b) Percent positive and MFI of MHC I expression from G2 and G3 populations. *** = p<0.001.

Surface expression of MHC I from flight mice or ground controls demonstrated two distinct populations. One population exhibited low intensity of MHC I expression that is well confined to G3. The other population had a diverse intensity range of MHC I expression that was generally higher compared to those in G2 (Fig 7a). Analysis of MHC I allowed grouping into MHC I^lo^ (G3) and MHC I^hi^ (G2) populations. In percent population analysis of MHC I expression, no differences were observed for any of the TLR agonist conditions between splenocytes isolated from flight mice or ground controls. Interestingly, there was an across the board decrease of MHC I MFI from splenocytes isolated from flight mice compared to ground controls in the G2 (MHC I^hi^) population. This trend was completely reversed in the G3 (MHC I^lo^) population in MHC I MFI, with splenocytes from flight mice demonstrating a significant increase in expression intensity compared to ground controls (Fig 7b). No differences were observed in expression of MHC II between flight mice and ground controls (data not shown).

**Fig 8:**
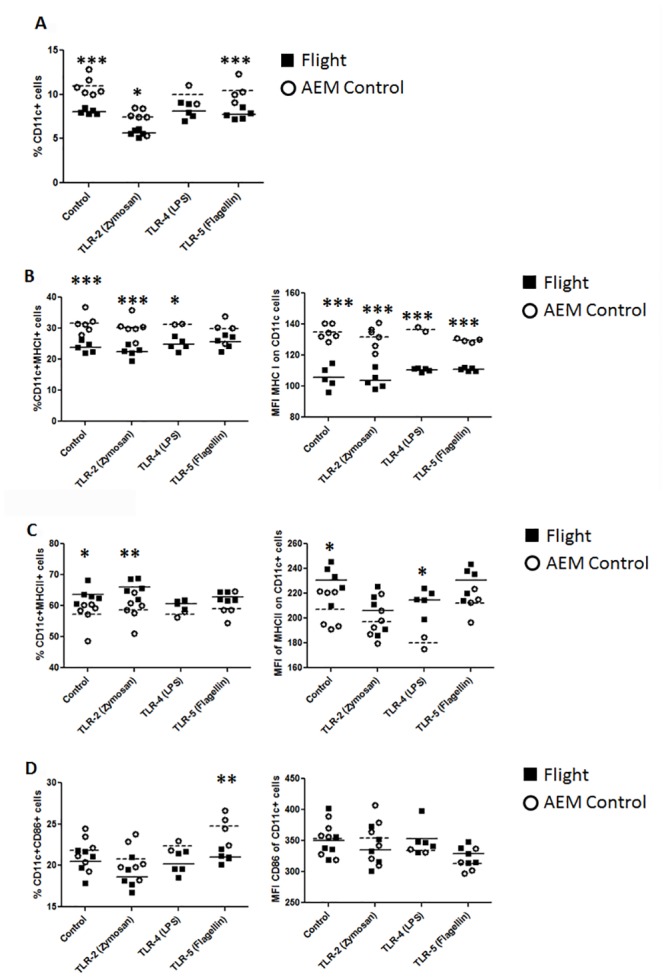
Dendritic cell populations changes after TLR stimulation in post-spaceflight mice. Flight (solid square) or ground AEM control (open circle) splenocytes were cultured in DMEM with 10% FBS with or without TLR agonists for 24 hours. Cells were collected and stained for expression of CD11c, MHC I, MHC II, CD86, and CD80. (8a) Total percent of dendritic cells (CD11c) of post-spaceflight or ground AEM control mice under various TLR stimulation conditions. (8b) Percent and MFI of dendritic cells expressing MHC I. (8c) Percent and MFI of dendritic cells expressing MHC II. 8d) Percent and MFI of dendritic cells expressing CD86. *** = p<0.001 **p = <0.01 * = p<0.05.

Expression of CD11c, a mouse dendritic cell marker, showed that splenocytes from flight mice had a global decrease in expression of CD11c positive cells under all culture conditions (Fig 8a). These dendritic cell populations were investigated for expression of antigen presentation and co-stimulatory molecules. CD11c^+^MCHI^+^ cells were decreased in both percent population and intensity of MHC I expression in all culture conditions in flight mice splenocytes compared to ground controls (Fig 8b). CD11c^+^MHCII^+^ splenocytes from flight mice demonstrated increased in percent population and intensity of MHC II expression compared to ground controls (Fig 8c). There was little difference between flight mice and ground controls in CD11c^+^CD86^+^ splenocytes, except a significant decrease in CD11c^+^CD86^+^ cells under TLR-5 stimulation (Fig 8d). Finally, there was very low overall expression of CD80 (data not shown).

### Cytokine Production from Stimulated Splenocytes Post-Spaceflight

The changes in surface expression of T-cell activation molecules, as well as dendritic cell antigen presentation and co-stimulatory molecules, during stimulation indicated potential functional dysregulation caused by spaceflight. Cytokine production during stimulation is often used to determine cell activity. Supernatants from splenocytes stimulated with T-cell stimulants or TLR agonists were further examined for cytokine production by ELISA.

Stimulation with ConA demonstrated a non-significant trend of increased IFN-γ production and a concurrent decrease in IL-2 production from flight mice splenocytes compared to ground controls (Table 3). TLR-2 and TLR-4 agonists also stimulated an increase in production of TNF-α and IL-6 from splenocytes isolated from flight mice versus ground controls. Only the increase in TNF-α induced by TLR-2 agonist represented a significant difference (Table 4). Little (below the level of detection of the assay) to no TNF-α and IL-6 were produced when stimulated with TLR-5 agonist (data not shown).

**Table 3:** IFN-γ and IL-2 Cytokine Production from Con-A Stimulated Splenocytes Post-Spaceflight.

	ConA		p value
	AEM Control	Flight	
**IFN-γ**	34.42 (4.013)	101.1 (75.56)	0.399
**IL-2**	185.2 (29.58)	110.4 (29.8)	0.1054

Data obtained by ELISA, from ConA (2μg/ml) stimulated splenocytes from post-spaceflight (Flight) mice or animal enclosure module (AEM) ground control mice. Average values given with standard deviation.

**Table 4:** TNF-α and IL-6 Cytokine Production from TLR-2 and TLR-4 Stimulated Splenocytes Post-Spaceflight.

	TLR-2		p value	TLR-4		p value
	AEM Control	Flight		AEM Control	Flight	
**TNF-α**	**44.33 (6.529)**	**80.83 (12.94)**	**0.0304**	48 (2.754)	73.7 (11.88)	0.1586
**IL-6**	11.92 (5.32)	12.42 (3.241)	0.9376	35.33 (2.603)	64.9 (22.16)	0.3565

Data obtained by ELISA, from Zymosan (TLR-2 agonist; 10μg/ml) or LPS (TLR-4 agonist; 200ng/mL) stimulated splenocytes from post-spaceflight (Flight) mice or animal enclosure module (AEM) ground control mice. Average values given with standard deviation.

## Discussion

The immune dysfunction post-spaceflight is well documented. This disruption in immune activity is believed to be partly responsible for observed spaceflight and post-flight related incidence of immune-related adverse health events that may occur during spaceflight. With future plans for longer duration space travel, the question of how changes in host immunity would affect overall crew health needs to be addressed. The experiments conducted in this report focused on changes that bridge innate and adaptive immune responses. This bridge is crucial to generating long-term memory protection against infection, and critical for mounting immune responses during reinfection and reactivation post flight. Specifically, this report investigated the ability of dendritic cells (DC) to respond to stimulation through TLRs, and T-cell responsiveness through the T-cell receptor.

To determine baseline receptor expression, the splenocytes were stained upon landing. These native expressions found no differences observed in the percent population changes of CD4 or CD8 T cells, with results reflecting similar findings from published works showing no changes in percent population of CD4 or CD8 T cells in rodents post-spaceflight [32–34]. Other reports show mixed changes. Specifically, some have identified increases in CD4 and CD8 populations [35–38], others show decreased CD4 and CD8 populations [39, 40], and others have shown a combination thereof [10] post-flight. Pecaut, et al. demonstrated that there was indeed a shift in leukocyte phenotypes to different organs, which may affect localization of response [41]. Likewise, conflicting reports are found from peripheral blood of astronauts post-flight [15, 16, 18, 20]. Some of these differences were attributed to the type of lymph organ examined, as thymus cells usually shows no change in CD4 and CD8 T cells populations while splenocytes do exhibit differences between flight animals and ground controls [36]. The seemingly contradictory evidence could be explained by different gating strategies used to determine percent positivity of events. The study presented here showed that splenocytes from mice post-flight demonstrated an increase in a cell population with high percentages of CD4^+^ and CD8^+^ cells.

While previous reports only examined percent positive populations, this study included examination of marker intensity, which was interpreted to indicate an increase in marker expression per cell. There was a significant decrease in the intensity of CD4 and an increase in intensity of CD8 expression on a per cell basis. Since CD4 and CD8 molecules play a role during T-cell recognition of antigen presentation by binding to the antigen presentation molecules, MHC I, MHC II, or CD1, any change in their surface expression could affect the avidity potential to alter T-cell stimulation. Indeed, the avidity/affinity model of peripheral T-cell regulation suggests that changes in CD4 and CD8 expression affect T-cell activation. CD8^lo^ cells require higher avidity of T-cell receptor and MHC I interactions for activation [42] and control/suppress other CD8 T-cell responsiveness to antigen [43].

This study also found splenocytes from flight mice had a significant decrease in intensity of CD25 expression on CD4^+^ and CD8^+^ T-cells compared to ground controls. CD25 is the IL-2 receptor, which is often used as a marker to denote regulatory T-cells. However, this decrease in CD25 intensity is not interpreted to indicate a change in regulatory T-cell post-flight, especially because the population of CD25^+^ splenocytes did not change. The CD25 ligand, IL-2, is the T-cell proliferating factor. Experiments on the Jurkat T cell line post-flight demonstrated a lack of proliferation and elevations of Fas/APO-1, suggesting spaceflight conditions induced apoptosis in T-cells [44, 45]. The decrease in CD25 expression on a per cell basis suggests that flight splenocytes downregulate factors for proliferation. And it has been clearly demonstrated that immediate early genes of lymphocytes are affected under conditions of microgravity [46].

Dendritic cells are the primary antigen presenting cells to bridge innate and adaptive immunity. DCs respond to invading pathogens, prime and activate antigen specific T-cells, and promote development of antigen specific memory. In the mouse, expression of CD11c, a β2 integrin, is a marker for DC identification. Splenocytes from flight mice demonstrated a significant increase in CD11c^+^ population compared to ground controls, suggesting an enhancement of the DC population. This is the first reported change of spaceflight on DC population dynamics. Interestingly, previous published reports examining another β2 integrin, CD11b, showed a decrease population change, interpreted as a decrease in monocytes/macrophages [40], which was also observed using CD14/16 markers for identification [10]. As with T-cells, spaceflight conditions were also reported to increased monocyte/macrophage population [15, 16]. Similar to T-cell subsets, macrophages and dendritic cells populations have differential proliferation results in response to spaceflight.

Examination of MHC I and II presentation molecule expression found that CD11c^+^ splenocytes post-flight had a significant increase in MHC I^+^ and a significant decrease in MHC II^+^ populations compared to ground controls. There are limited reports on antigen presentation molecule expression, and both published articles also found a decrease in class II presentation molecules expressing populations [47, 48]. These results confirm that spaceflight alters class II presentation molecule expression, which may indicate a diminution in the potential to effectively present antigens to CD4^+^ T-cells.

Splenocytes post-spaceflight demonstrated an increase in bead uptake compared to ground controls. This is the first report that flight conditions influence particle uptake. The implications suggest that spaceflight does not hinder particle uptake, and may even enhance the function. However, it is not clear how alteration of particulate uptake would affect host immune status. Limited hindlimb suspension model studies may shed light on this, if one takes the assumption that uptake of pathogenic organisms are similar to that of beads. In those studies, *in vivo* infection found no change in clearance of rotavirus [49] and a decrease in bacterial clearance of *Pseudomonas aeruginosa* [50] and *Klebsiella pneumoniae* [51]. This suggests that other immune effects resulting from spaceflight contribute to clearance dysfunction, even if there are increased uptake events of particulates. Overall, this is difficult to assess, and we realize that beads travel along different phagocytic pathways than the agents mentioned.

Development of effective immunity towards pathogens relies on responding to innate signals. The toll-like receptors (TLR) are well established pattern recognition receptors stimulated by pathogenic motifs [52]. The most commonly used TLR ligand to investigate spaceflight effect on cell function is lipopolysaccharide (LPS). In humans, blood cells isolated during spaceflight responded to LPS by increasing production of IL-8, however, this finding resolved upon post-spaceflight examination [18]. LPS has also shown to promote lower production of IL-6, TNF-α, and IL-10 and increased IL-1β post-spaceflight in humans [47]. Reports in mice show a mixed response when looking a whole splenocyte populations [53]. Purified macrophages flow in space demonstrated an increase in both IL-1 and TNF-α production in response to LPS [54]. While that report did not observe a significant increase in inflammatory cytokines upon LPS stimulation, the flight splenocytes did exhibit a trend of increase in TNF-α and IL-6 production compared to ground controls. The data presented here extended observations to include cytokine production with the TLR-2/6 and TLR-5 ligands, zymosan (fungal) and flagellin (bacterial) respectively. Zymosan stimulated a significant increase in TNF-α; no cytokine changes were observed upon flagellin stimulation. Chapes et al. investigated stimulation with poly I:C, a TLR-3 ligand, and found an increase in IFN-α [54]. For the most part, flight exposure did not have a deleterious effect on TLR activation and stimulation, suggesting that the innate immune inflammatory response remains intact.

Few published articles have examined changes in surface expression of presentation and co-stimulatory molecules after TLR stimulation. While cytokine production is an important indicator of immune response, the main effect of how DCs response to TLR stimulation is to increase antigen presentation and stimulate T-cells [55]. Splenocytes demonstrated similar changes DC surface molecule expression post flight, regardless of TLR stimulation. For example, the DC population from the flight mice had altered MHC I and MHC II expressing populations compared to ground controls, even in cultures that did not receive TLR-agonist stimulation.

Regarding adaptive immune functions, T-cells are critical for control of re-infection and re-activation events. Unlike DCs, the effect post-spaceflight on T-cell activity has been well documented. Stimulating with anti-CD3/CD28, which simulates T-cell activation by antigen presenting cells, resulted in increased IFN-γ and decreased IL-2 [18, 56]. ConA stimulation, which stimulates T-cells through the T-cell receptor without requiring the secondary signal (CD28), resulted in no change or increased IL-2 [36, 57], increased IL-3, and decreased IFN-γ [58]. Use of other mitogens, such as PHA or PMA/Ionomycin, that stimulates by bypassing both the T-cell and co-stimulatory receptors, led to no change or decreased IL-2 [10, 57], decreased IFN-γ [10] and decreased TNF-α [18]. The data in this report reflects some differences in reported observations, although in line with that reported by Gridley, et al. [59]. Rather, the results reported here for ConA stimulation demonstrated a non-significant increase in IFN-γ and decreased IL-2. The discrepancy in cytokine results could be differences in cell culture number, concentration of stimulants, and time of stimulation.

Surface molecule expression was examined in stimulated T-cells. Little or no differences were observed in the flight mice versus ground controls, with two notable exceptions. Regardless of stimulation parameters, the cultured splenocytes isolated from flight mice were significantly depressed in their expression of CD4. Second, while expression of CD8 intensity immediately post-spaceflight demonstrated an increase on a per cell basis, further *in vitro* culture of cells distinctively identified two sets of CD8 cells, CD8^lo^ and CD8^hi^. This distinction disappeared upon ConA stimulation. Indeed, the flight mice splenocytes evidenced a significant increase in CD4 or CD8 T-cells that also expressed CD25 or CD25/28. Therefore, it is important for future studies to define gating strategies, as we have done here. We suggest that CD25, along with CD69, are used as markers of early T-cell activation. Short-term spaceflight increased expression of CD25/69 when stimulated with anti-CD3/28 [20]. The results with ConA may indicate that flight mice could demonstrate increased early T-cell activation upon stimulation, compared to ground controls.

Our report focused on phenotypic alterations that bridge innate and adaptive immune responses crucial for generating protective immune responses during reinfection and reactivation post flight. It would indeed be important to understand how long the phenotypic changes were sustained post flight; analysis of genetic alterations or changes in methylation status would certainly be warranted. The condition of the spaceflight environment has been shown to alter cytosine methylation patterns to generate heritable epigenetic changes in rice. Both hypo- and hypermethylation events can occur, with concurrent dysregulation of gene expression. Genes for DNA methyltransferases were very sensitive to spaceflight conditions [60, 61]. Similar effects were identified in microgravity studies in human lymphocytes [62, 63], where both DNA methylation genes, as well as histone acetylation genes, were affected. In addition, DNA repair mechanisms were also diminished. Overall this suggests both an accumulation of damaged DNA as well as the potential for inheritable and sustainable changes in cellular phenotypic activity.

In conclusion, there exists a large body of evidence that spaceflight does impact T-cell activity and cause global suppression in response to multiple stimulants. Indeed, these “type IV hypersensitive” class responses have been found to be reduced to a variety of antigens during and post spaceflight [14, 64]. The data from this study shed additional light towards molecular mechanisms involved in immune changes induced by spaceflight that could alter activation of innate and adaptive immunity, at least post flight. It remains to be determined if these changes may also impact responses during active flight. Given the limited number of subjects for spaceflight studies, future analysis should continue to examine both innate (DC) and adaptive (T-cell) responses.

## Supporting Information

S1 File (PPTX)
