# Supplementary material for: Post-Spaceflight (STS-135) Mouse Splenocytes Demonstrate Altered Activation Properties and Surface Molecule Expression
Source: PLoS One. 2015 May 13;10(5):e0124380. doi: 10.1371/journal.pone.0124380 (PMC4430214; doi:10.1371/journal.pone.0124380)
Supplement: S1 File — (PPTX) [file pone.0124380.s001.pptx]

## Slide 1
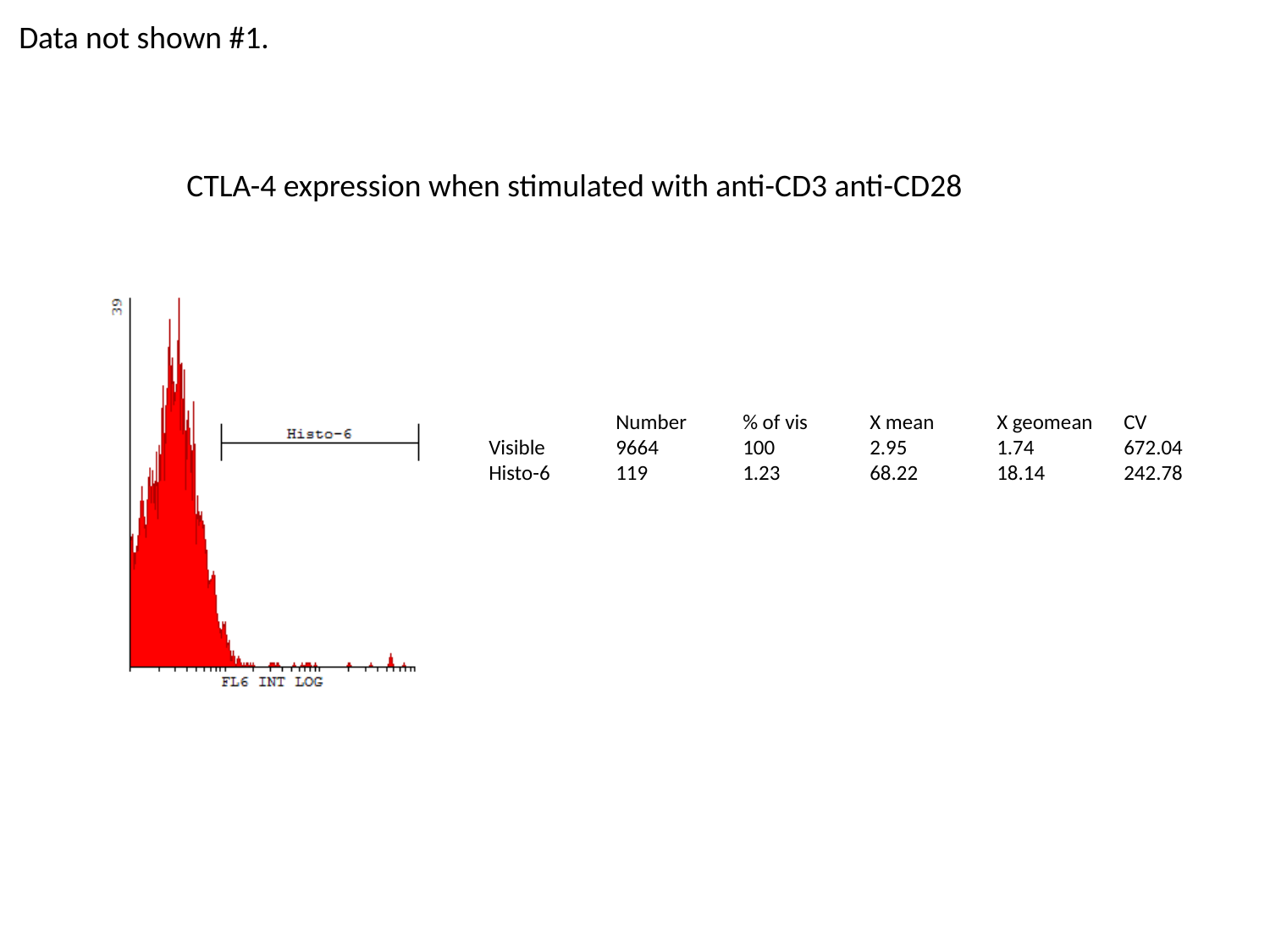

Data not shown #1.
CTLA-4 expression when stimulated with anti-CD3 anti-CD28
	Number	% of vis	X mean	X geomean	CV
Visible	9664	100	2.95	1.74	672.04
Histo-6	119	1.23	68.22	18.14	242.78

## Slide 2
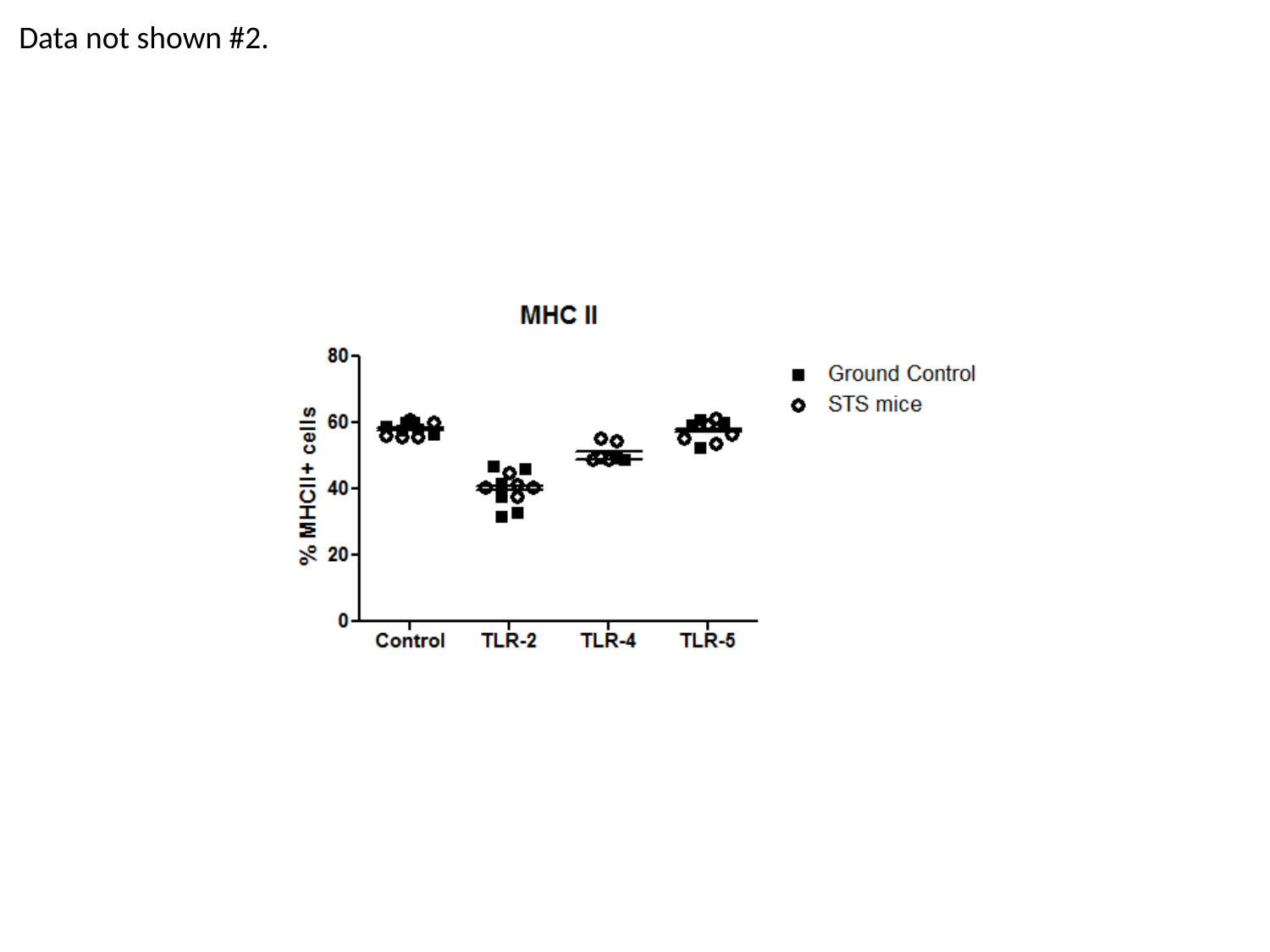

Data not shown #2.

## Slide 3
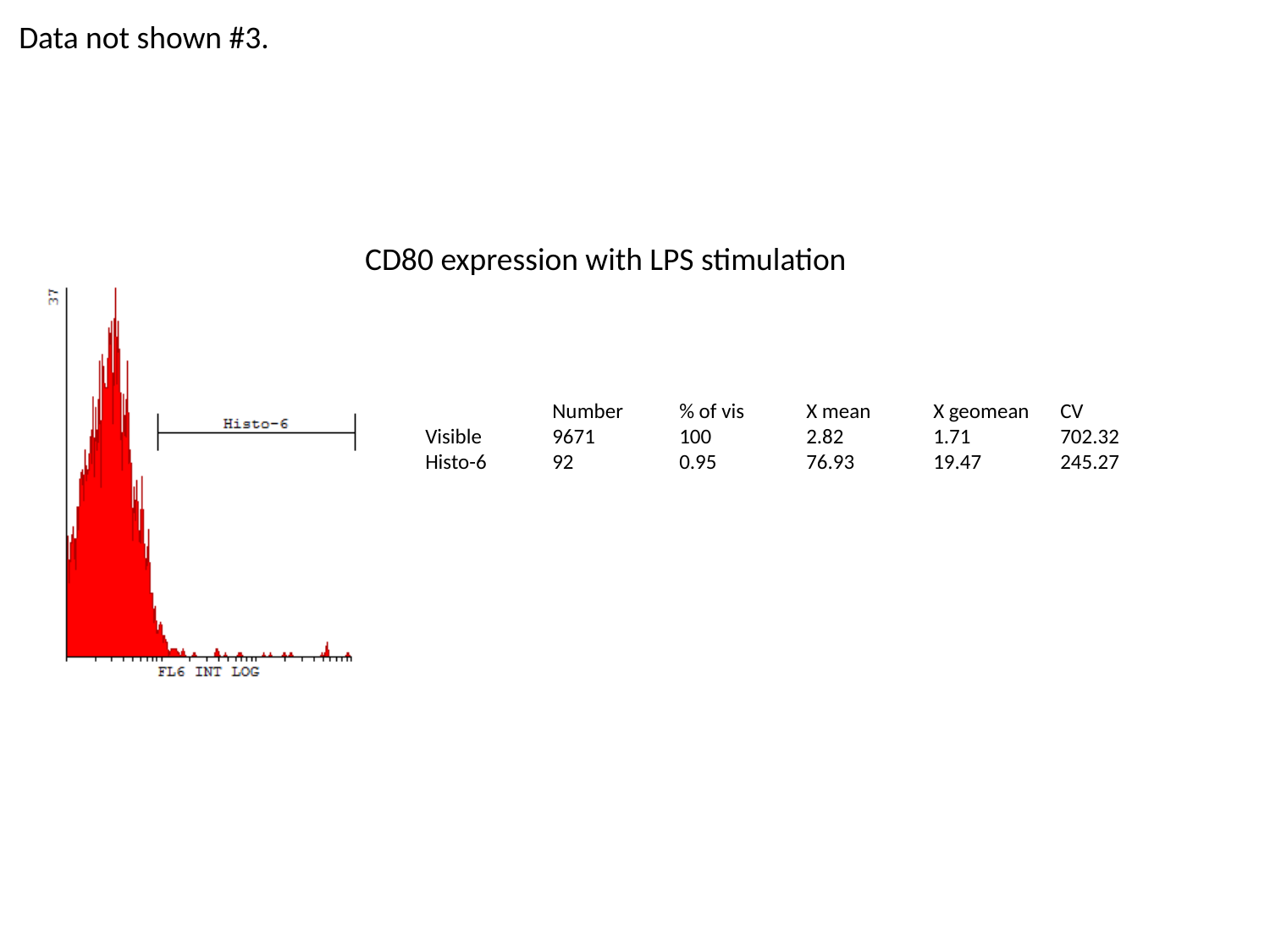

Data not shown #3.
CD80 expression with LPS stimulation
	Number	% of vis	X mean	X geomean	CV
Visible	9671	100	2.82	1.71	702.32
Histo-6	92	0.95	76.93	19.47	245.27

## Slide 4
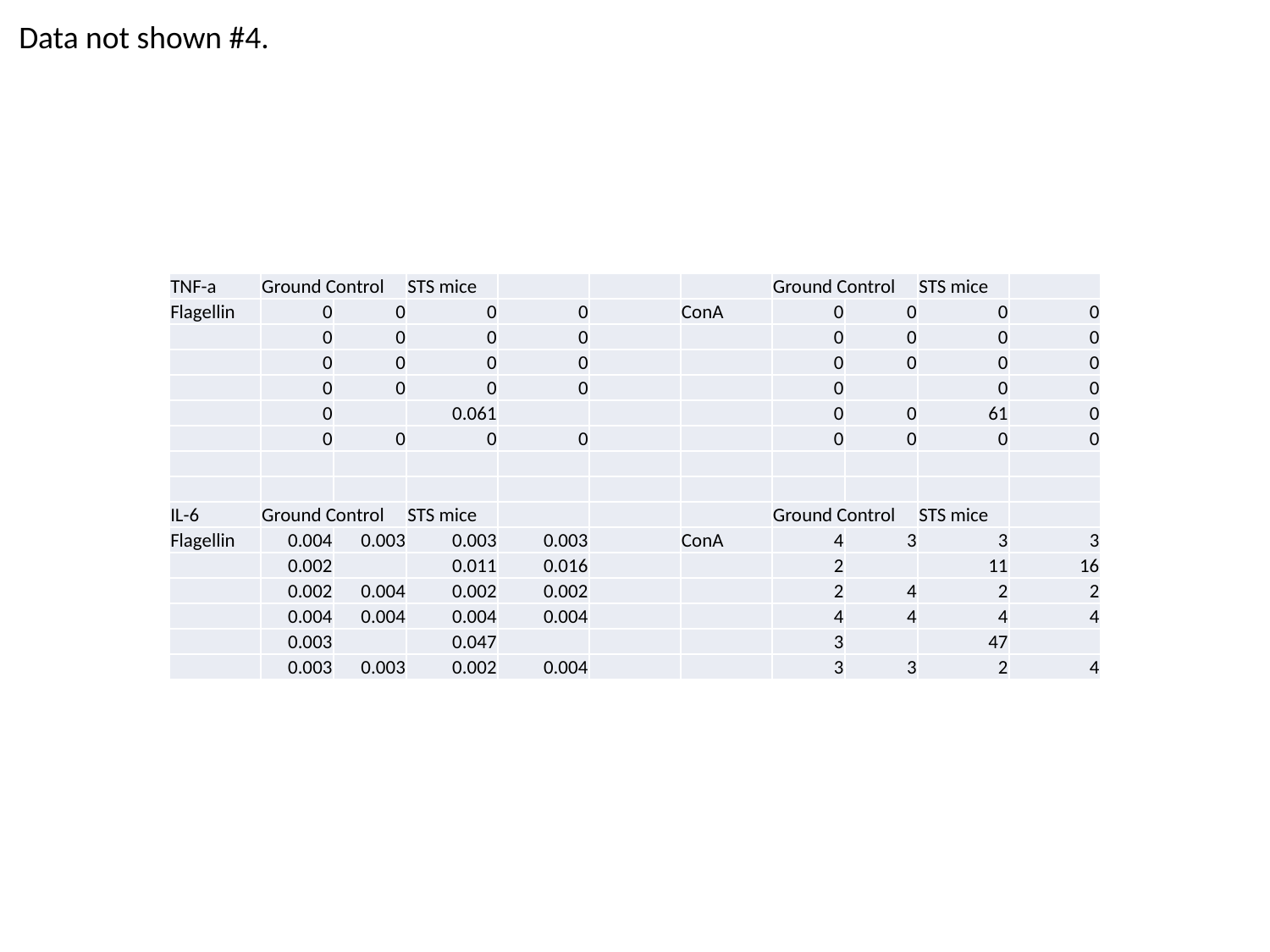

Data not shown #4.
| TNF-a | Ground Control | | STS mice | | | | Ground Control | | STS mice | |
| --- | --- | --- | --- | --- | --- | --- | --- | --- | --- | --- |
| Flagellin | 0 | 0 | 0 | 0 | | ConA | 0 | 0 | 0 | 0 |
| | 0 | 0 | 0 | 0 | | | 0 | 0 | 0 | 0 |
| | 0 | 0 | 0 | 0 | | | 0 | 0 | 0 | 0 |
| | 0 | 0 | 0 | 0 | | | 0 | | 0 | 0 |
| | 0 | | 0.061 | | | | 0 | 0 | 61 | 0 |
| | 0 | 0 | 0 | 0 | | | 0 | 0 | 0 | 0 |
| | | | | | | | | | | |
| | | | | | | | | | | |
| IL-6 | Ground Control | | STS mice | | | | Ground Control | | STS mice | |
| Flagellin | 0.004 | 0.003 | 0.003 | 0.003 | | ConA | 4 | 3 | 3 | 3 |
| | 0.002 | | 0.011 | 0.016 | | | 2 | | 11 | 16 |
| | 0.002 | 0.004 | 0.002 | 0.002 | | | 2 | 4 | 2 | 2 |
| | 0.004 | 0.004 | 0.004 | 0.004 | | | 4 | 4 | 4 | 4 |
| | 0.003 | | 0.047 | | | | 3 | | 47 | |
| | 0.003 | 0.003 | 0.002 | 0.004 | | | 3 | 3 | 2 | 4 |
